# Mechanistic insights into adipose-derived stem cells and exosomes in ischemia-reperfusion injury repair: from shared pathways to organ-specific therapeutics

**DOI:** 10.3389/fcell.2025.1621289

**Published:** 2025-06-20

**Authors:** Jiaqian Si, Jie Wang, Hao Dai, Tuochen Lv, Songyun Zhao, Wanying Chen, Liqun Li, Siqi Ding, Yucang He

**Affiliations:** ^1^ Department of Plastic Surgery, First Affiliated Hospital of Wenzhou Medical University, Wenzhou, Zhejiang, China; ^2^ Department of Neurology, The Affiliated Yiwu hospital of Wenzhou Medical University, Yiwu, Zhejiang, China

**Keywords:** adipose-derived stem cells, exosomes, ischemia-reperfusion injury, multimechanism synergy, therapeutic strategy

## Abstract

Ischemia-reperfusion injury (IRI) has become a significant challenge for clinical treatment due to the complex multi-mechanism pathological cascade response, including oxidative stress, inflammatory bursts, and programmed cell death. Adipose-derived stem cells (ADSCs) and their exosomes (ADSCs-exosomes) are emerging as a breakthrough therapeutic strategy to reverse IRI, owing to their multi-target synergistic effects. This review systematically analyzes the two major repair modes of ADSCs and ADSCs-exosomes: the “common protection” mechanism, which includes anti-inflammatory, anti-oxidative, and anti-apoptotic effects through paracrine regulation of miRNAs targeting the NF-κB/NRF2/β-catenin signaling axis; and precision repair, which is achieved through organ-specific targets, including hepatic mitochondrial dynamics and pyroptosis inhibition, cardiac macrophage polarization and neutrophil clearance, renal anti-fibrosis and erythropoietin (EPO) activation, as well as brain iron death regulation and microglial remodeling. From the perspective of the mechanism interaction network, this paper first proposes a theoretical framework of “multi-organ shared core pathways and dynamic regulation of different targets.” It also reviews the translational potential of combined therapeutic strategies based on engineered exosomes delivery systems and biomaterials, emphasizing the optimization of delivery efficiency and functional enhancement to address the bottleneck of clinical applications. The ADSCs-mediated IRI intervention system provides an essential theoretical and technical basis for the development of individualized precision therapies.

## 1 Introduction

IRI is a pathological process wherein restoration of blood flow exacerbates damage to ischemic tissues. Its underlying mechanisms involve multifaceted biological processes, including reactive oxygen species (ROS) generation, inflammatory response activation, and apoptosis induction. During ischemia, insufficient oxygen supply depletes ATP, leading to cellular stress. Upon reperfusion, excessive ROS trigger lipid peroxidation, compromising cell membrane integrity and function. Additionally, IRI activates the complement and coagulation systems, releasing inflammatory mediators that promote neutrophil infiltration. These neutrophils release further ROS and proteases, amplifying tissue damage. Mitochondrial dysfunction during IRI releases cytochrome C, activating caspase family proteins and inducing apoptosis. These interconnected pathways collectively drive IRI progression (Zhang et al., 2024). Clinically, IRI manifests in conditions such as organ transplantation, myocardial infarction, stroke, and limb injuries (Capuzzimati et al., 2022; Hausenloy and Yellon, 2013; Liu and Man, 2023; Liu et al., 2021; Przykaza, 2021; Zhao et al., 2018). IRI poses significant threats to individual health and burdens healthcare systems. Current pharmacologic and nonpharmacologic therapies for IRI (including surgical techniques and gas therapy) are limited in effectiveness and often fail to completely reverse the injury, underscoring the need for novel therapeutic strategies (Wang et al., 2021). For example, NO, a heme oxygenase byproduct, is used clinically to treat ischemia-reperfusion injury (IRI) through its vasodilatory, anti-apoptotic, anti-inflammatory, and anti-thrombotic effects. However, NO has a narrow therapeutic window (1–80 ppm), posing potential toxicity risks. Similarly, emerging pharmacological agents like curcumin face clinical limitations including poor solubility, low bioavailability, and inadequate absorption, restricting their therapeutic application for IRI (Naito et al., 2020; Redaelli et al., 2022).

Stem cell therapy, an advancing frontier in regenerative medicine, has garnered attention for its tissue repair and immunomodulatory potential (Wu et al., 2022; Yu et al., 2022). Stem cells secrete growth factors, cytokines, and exosomes to promote regeneration of damaged tissues (Xia et al., 2019). However, challenges such as immune rejection, tumorigenicity, and complex cell sourcing limit their clinical application (Herberts et al., 2011; Lukomska et al., 2019). In contrast, ADSCs and their exosomes offer distinct advantages for IRI treatment due to their regenerative capabilities. ADSCs are abundant, easily harvested, exhibit low immunogenicity (Frese et al., 2016; Kern et al., 2006; Si et al., 2019), and possess multidirectional differentiation potential (Zuk et al., 2002) and robust paracrine functions (Miana and González, 2018). ADSCs-exosomes, enriched with bioactive molecules such as proteins, lipids, and microRNAs (Pefanis et al., 2015; Wang et al., 2023a), mitigate IRI-induced damage by suppressing inflammation, reducing oxidative stress, inhibiting apoptosis, and promoting angiogenesis and tissue regeneration. This multifaceted therapeutic approach suggests that ADSCs and their exosomes may represent potential candidates for developing safer and more effective IRI treatments.

**FIGURE 1:**
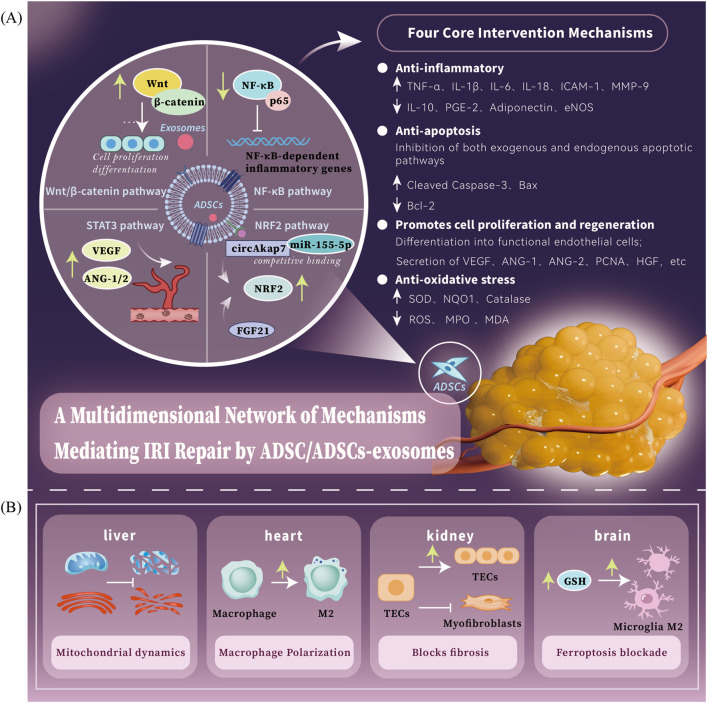
ADSCs/ADSCs-exosomes mediates a multidimensional network of mechanisms for IRI repair. **(A)** ADSCs/ADSCs-exosomes mainly act on four major pathways: the Wnt/β-catenin signaling pathway is a key regulator that promotes cell proliferation and tissue regeneration; the NF-κB signaling pathway is a key mediator that inhibits inflammatory responses; the STAT3 signaling pathway is a key factor that enhances the ability of neovascularization; the NRF2 signaling pathwayis an important target for anti-oxidative stress. The four core intervention directions of ADSCs therapy: anti-inflammation, anti-oxidation, anti-apoptosis and pro-regeneration. **(B)** ADSCs/ADSCs-exosomes have different core intervention strategies for different organs: heart - macrophage polarization, liver - mitochondrial dynamics, brain - iron death blockade, kidney - pathological fibrosis process blockade.

This review elucidates the core mechanisms by which ADSCs and their exosomes contribute to IRI repair (Figure 1A). It summarizes recent advances in their therapeutic potential, applications, and benefits across various organs, based on current literature (Figure 1B). Current investigations into the therapeutic potential of ADSCs in IRI remain predominantly confined to preclinical animal models, with no clinical trials yet reported. Consequently, the experimental findings presented in this review are exclusively derived from animal-based research. These preclinical data establish a critical foundation for future translational studies and potential clinical applications. Additionally, we address limitations of standalone ADSCs transplantation and explore strategies to enhance its efficacy.

## 2 Functional analysis of ADSCs and exosomes: core mechanisms of dual-pathway synergistic repair of IRI

### 2.1 Direct effects

#### 2.1.1 Multidirectional differentiation potential: molecular basis of cell replacement

IRI triggers a cascade of pathological events, including ROS bursts and mitochondrial dysfunction, leading to irreversible damage such as synaptic disintegration and microvillus shedding in target cells like neurons and renal tubular epithelial cells. ADSCs exhibit multidirectional differentiation potential, enabling them to address this damage by replacing injured cells. Under specific induction conditions, ADSCs can differentiate into diverse cell types, including osteoblasts, chondrocytes, adipocytes, and cardiomyocytes (Orbay et al., 2012). In the IRI microenvironment, ADSCs can directionally differentiate into organ-specific cells, such as neuron-like cells, renal tubular epithelial-like cells, and endothelial-like cells, providing a functional cellular reserve for structural repair (Gao et al., 2012; Wang et al., 2022). This differentiation capacity is governed by complex molecular regulatory mechanisms, encompassing transcription factors, signaling pathways, and epigenetic modifications. For instance, during osteogenic differentiation, upregulation of transcription factors Runx2 and Osterix drives expression of osteogenesis-related genes, promoting ADSCs differentiation into osteoblasts (Li et al., 2024). The Wnt/β-catenin signaling pathway also plays a pivotal role in ADSCs differentiation. Activation of this pathway enhances osteogenic differentiation, while its inhibition promotes adipogenic differentiation (Matsushita et al., 2020). Furthermore, epigenetic modifications, including DNA methylation and histone modifications, regulate ADSCs differentiation fate by altering chromatin structure and gene accessibility, thereby influencing the expression of differentiation-associated genes (Daniunaite et al., 2015).

#### 2.1.2 Pro-angiogenic strategies: dynamic regulation of microcirculatory perfusion

ADSCs orchestrate neovascularization through direct differentiation and paracrine signaling, synergistically enhancing microcirculatory perfusion. ADSCs can differentiate into functional endothelial cells, directly contributing to angiogenesis (Uysal et al., 2009). They also regulate endothelial function via cell-to-cell interactions, promoting vascular maturation and stabilization. Additionally, ADSCs-exosomes deliver microRNAs and bioactive molecules to endothelial cells, modulating their proliferation, migration, and expression of angiogenesis-related genes (Zhou et al., 2024). Paracrine mechanisms further amplify ADSCs-mediated angiogenesis. ADSCs secrete pro-angiogenic factors, including vascular endothelial growth factor (VEGF), angiopoietin-1, angiopoietin-2, basic fibroblast growth factor, and endothelial nitric oxide synthase, which collectively enhance blood flow reconstruction in ischemic tissues (Gao et al., 2012; Jiao et al., 2019; Zhou et al., 2020; 2017). Concurrently, ADSCs produce anti-angiogenic factors, such as pigment epithelium-derived factor, to mitigate pathological vascular abnormalities and leakage (Leong et al., 2020). Activation of the IL-6/STAT3 signaling pathway further regulates angiogenesis-related gene expression, promoting vascular network normalization (Pu et al., 2017). These strategies significantly increase microvessel density, improve tissue oxygenation, facilitate metabolic waste removal, and elevate ATP levels. However, achieving optimal spatiotemporal regulation of pro-angiogenic and anti-leakage factors (e.g., VEGF/PEDF balance) requires further refinement, potentially through gene editing or biomaterial-based delivery systems.

#### 2.1.3 Direct inhibition of pyroptotic inflammasome components

Pyroptosis, an inflammatory form of programmed cell death, significantly contributes to IRI. ADSCs and their exosomes directly suppress pyroptotic pathways, mitigating inflammatory responses and cellular damage. ADSCs-secreted factors inhibit the activation of the NLRP3 inflammasome, reducing caspase-1 cleavage and activation, which in turn prevents gasdermin D (GSDMD) cleavage and pyroptosis onset (Yang and Zhang, 2024). This mechanism suppresses the release of pro-inflammatory cytokines interleukin-1β (IL-1β) and interleukin-18 (IL-18), key mediators of pyroptosis, thereby interrupting the cascade amplification of inflammatory signals (Wang et al., 2023b). However, the precise molecular pathways, such as NLRP3/AIM2-ASC-pro-caspase-1 or TLR4/MyD88/NF-κB, and targeted delivery strategies for ADSCs in regulating pyroptosis remain to be fully elucidated. Spatiotemporal omics and functional knockout experiments are needed to resolve these mechanisms.

#### 2.1.4 Direct intervention in ferroptosis key enzyme activities

ADSCs and their exosomes alleviate IRI by directly modulating ferroptosis, an iron-dependent programmed cell death characterized by lipid peroxidation, glutathione (GSH) depletion, and glutathione peroxidase 4 (GPX4) inactivation. Key mechanisms include: (1) upregulating GPX4 expression via ADSCs-exosomes to convert toxic lipid peroxides into non-toxic lipid alcohols, thereby suppressing ferroptosis in inflammatory conditions (e.g., sepsis); and (2) restoring GSH homeostasis by enhancing GSH biosynthesis through destabilizing ATF3 mRNA (reducing ATF3-mediated inhibition of System Xc^−^ and SLC7A11) and downregulating CHAC1 expression, which mitigates GSH degradation. These synergistic actions inhibit ferroptosis-driven cellular damage, offering targeted therapeutic strategies for IRI (Jin et al., 2024; Shen et al., 2023; Bersuker et al., 2019; Wang et al., 2024).

### 2.2 Indirect effects

#### 2.2.1 Inflammatory remodeling: from NF-κB regulation to macrophage polarization

ADSCs and their secreted exosomes ameliorate IRI by dual modulation of the nuclear factor kappa B (NF-κB) pathway, a central regulator of inflammation that drives the transcription of pro-inflammatory cytokines, such as tumor necrosis factor-alpha (TNF-α), IL-1β, and interleukin-6 (IL-6), through IκB kinase (IKK)-induced phosphorylation and degradation of IκBα, which enables nuclear translocation of the p65/p50 heterodimer (Yu et al., 2020). ADSCs suppress inflammatory responses by selectively inhibiting both the expression and phosphorylation of the p65 subunit, thereby reducing levels of pro-inflammatory mediators (TNF-α, IL-1β, ICAM-1, MMP-9) while concurrently enhancing anti-inflammatory repair mechanisms via upregulation of interleukin-10 (IL-10), prostaglandin E2 (PGE2), and adiponectin, coupled with improved endothelial function through endothelial nitric oxide synthase (eNOS) phosphorylation (Chen et al., 2013; Jiao et al., 2019; Takamura et al., 2016; Piao et al., 2022). Furthermore, ADSCs reprogram macrophage polarization by shifting the balance from pro-inflammatory M1 to anti-inflammatory M2 phenotypes, potentially mediated by the IL-10/STAT3 signaling axis (Liu et al., 2024; Ishiuchi et al., 2023; Zhang et al., 2020). However, challenges persist in optimizing inflammatory remodeling, particularly in achieving spatiotemporal precision in NF-κB inhibition and anti-inflammatory factor secretion to avoid immunosuppression, as well as in understanding microenvironment-dependent macrophage polarization dynamics (M1/M2 ratio), which are influenced by oxygen gradients and metabolic alterations—complexities that necessitate integrated approaches combining metabolomics and single-cell sequencing technologies.

#### 2.2.2 Apoptosis inhibition: dual protective effects of ADSCs targeting the Bcl-2/Bax pathway

ADSCs mitigate apoptosis in IRI by modulating apoptotic signaling pathways, thereby preserving tissue function. ADSCs exert dual regulatory effects on both extrinsic (Fas/FasL/FADD/pro-caspase-8) and intrinsic (CytC/dATP/APAF1/pro-caspase-9) apoptotic pathways. These interventions significantly reduce the expression of pro-apoptotic factors, such as cleaved caspase-3 and Bax, while upregulating anti-apoptotic proteins, including Bcl-2. This dual mechanism decreases the proportion of apoptotic cells in target organs following IRI (Cheng et al., 2022; Gao et al., 2012; Wang et al., 2023a; Zhou et al., 2020). Additionally, ADSCs-exosomes attenuate cellular damage by activating the extracellular signal-regulated kinase 1/2 (ERK1/2)-glycogen synthase kinase-3β (GSK-3β) pathway, which further modulates the Bcl-2/Bax ratio (Zhang et al., 2021).

#### 2.2.3 Regenerative repair: ADSCs-mediated paracrine-Wnt/β-catenin Cross-pathway regulation

In IRI, ADSCs promote tissue regeneration by synergistically modulating pro-proliferative factors and key signaling pathways. ADSCs enhance the expression of regeneration-associated factors, including hepatocyte growth factor (HGF), cyclin D1, and proliferating cell nuclear antigen (PCNA), which drive cell proliferation. Concurrently, they counteract the suppression of regenerative signaling in the inflammatory microenvironment by activating the Wnt/β-catenin pathway (Jiao et al., 2019; Piao et al., 2022). Mechanistic studies demonstrate that N-cadherin overexpression enhances regeneration efficiency through a dual mechanism: (1) improving ADSCs retention in injured tissues and (2) upregulating HGF expression via a β-catenin-dependent pathway. This “structural anchoring-paracrine” model significantly promotes cell proliferation, as evidenced in myocardial infarction models (Yan et al., 2020). Despite their potential to accelerate tissue repair, ADSCs face challenges in clinical translation: (1) regenerative signaling must be precisely tailored to organ-specific requirements, and (2) the targeting efficiency of exogenous ADSCs limits sustained regenerative effects. Future advancements should leverage single-cell spatial transcriptomics and synthetic biology to develop microenvironment-responsive delivery systems, enabling targeted initiation and dynamic regulation of regeneration programs.

#### 2.2.4 Redox homeostasis regulation: NRF2-mediated defense and ROS scavenging Synergy

ADSCs counteract IRI-induced oxidative stress, marked by ROS overproduction (e.g., superoxide anions and hydrogen peroxide) and resultant lipid peroxidation, DNA damage, and cellular dysfunction, through a dual “enzyme scavenging-transcriptional regulation” mechanism. First, ADSCs restore redox balance by enhancing superoxide dismutase (SOD) activity while reducing ROS, myeloperoxidase (MPO), and malondialdehyde (MDA) levels to directly neutralize oxidative toxicants (Ge et al., 2018; Zhang et al., 2021). Second, conditioned medium from differentiated ADSCs (HBAC-CM) activates a systemic NRF2-mediated antioxidant defense by facilitating fibroblast growth factor 21 (FGF21)-dependent nuclear translocation of nuclear factor erythroid 2-related factor 2 (NRF2), which triggers transcriptional upregulation of downstream targets, including NAD(P)H quinone dehydrogenase 1 (NQO1), sestrin 2 (SESN2), SOD1, and catalase, forming a comprehensive antioxidant network (Moon et al., 2022). Concurrently, ADSCs-exosomes deliver circular RNA Akap7 (circAkap7), which sequesters miR-155-5p via competitive binding, thereby alleviating miR-155-5p-induced suppression of NRF2 and amplifying antioxidant signaling (Xu et al., 2020). This synergistic interplay between enzymatic ROS scavenging and NRF2-driven transcriptional regulation provides a multifaceted strategy to mitigate oxidative tissue damage, highlighting clinically actionable targets for refining antioxidant therapies.

## 3 ADSCs and exosomes in organ IRI

### 3.1 Hepatic IRI: bidirectional regulation of mitochondrial homeostasis and pyroptosis inhibition

#### 3.1.1 Pathologic mechanisms of hepatic IRI

Hepatic IRI is characterized by a vicious cycle of mitochondrial dysfunction and NLRP3 inflammasome-mediated pyroptosis. During ischemia, excessive dynamin-related protein 1 (DRP1)-dependent mitochondrial fragmentation impairs ATP synthesis. Subsequent reperfusion triggers ROS bursts, promoting NLRP3 oligomerization. This leads to the formation of NLRP3-ASC-caspase-1 complexes, activating caspase-1. The activated inflammasome exacerbates injury through two mechanisms: (1) cleavage of GSDMD, inducing hepatocyte pyroptosis, and (2) maturation and release of pro-inflammatory cytokines IL-1β and IL-18 (Xu et al., 2020; Zhang et al., 2021). Additionally, downregulation of mitochondrial fusion proteins, such as mitofusin (MFN), in hepatocytes hinders mitochondrial network repair. Structural alterations in mitochondria facilitate the formation of the mitochondrial permeability transition pore (mPTP), triggering necrotic cell death and apoptosis, which contribute to organ dysfunction and metabolic abnormalities (Zhang et al., 2021). Concurrently, endoplasmic reticulum (ER) stress manifests as structural expansion and dysfunction, further aggravating hepatic injury (Jiao et al., 2020).

#### 3.1.2 Regulatory effects of ADSCs and exosomes

ADSCs and their exosomes alleviate hepatic IRI by targeting the mitochondria-inflammasome-endoplasmic reticulum (ER) axis through multifaceted mechanisms. ADSCs-exosomes regulate mitochondrial homeostasis by bidirectionally modulating mitochondrial dynamics proteins: enhancing fusion proteins optic atrophy 1 (OPA1) and mitofusins 1/2 (MFN1/2), while suppressing fission proteins dynamin-related protein 1 (DRP1) and fission 1 (FIS1) (Zhang et al., 2021). Concurrently, ADSCs-exosomes-derived PGE2 inhibits excessive mitochondrial permeability transition pore (mPTP) opening via glycogen synthase kinase-3β (GSK-3β) phosphorylation (Zhang et al., 2021). For ER stress (ERS) attenuation, ADSCs downregulate key ERS markers, including glucose-regulated protein 78 (GRP78), phosphorylated eukaryotic initiation factor 2α (p-eIF2α), activating transcription factor 6 (ATF6), and X-box binding protein 1 (XBP1), while suppressing apoptotic effectors such as phosphorylated c-Jun N-terminal kinase (p-JNK), activating transcription factor 4 (ATF4), and C/EBP homologous protein (CHOP) (Jiao et al., 2020). Furthermore, inflammasome and inflammatory pathway suppression is achieved by ADSCs-exosomes-mediated inhibition of the NLRP3-ASC-caspase-1 complex assembly, reducing pyroptosis-related proteins GSDMD, interleukin-1β (IL-1β), and IL-18 via blockade of the TLR4/MyD88/NF-κB signaling pathway (Wang et al., 2023b). These coordinated actions highlight the therapeutic potential of ADSCs and exosomes in mitigating hepatic IRI through integrated modulation of mitochondrial-ER-inflammasome crosstalk.

Notably, ADSCs and their exosomes exhibit distinct roles in regulating the pyroptosis pathway. Piao et al. (2022) reported that, despite ADSCs inhibiting NLRP3 inflammasome activation (evidenced by reduced ASC and cleaved caspase-1 expression), gasdermin D N-terminal (GSDMD-N) protein expression was unexpectedly upregulated. In contrast, ADSCs-exosomes significantly reduced the expression of pyroptosis-related proteins, including GSDMD-N, which contrasts with the anticipated inhibitory effect of ADSCs on pyroptosis. In contrast, treatment with ADSC-derived exosomes (ADSCs-Exo) markedly reduced the expression of multiple pyroptosis-related proteins, including GSDMD-N, indicating a complex regulatory mechanism underlying the roles of ADSCs and ADSCs-Exo in modulating cellular pyroptosis. Potential contributing factors include cell type-specific responses—such as differential reactions of macrophages versus hepatocytes to ADSC intervention—and mechanistic disparities between ADSCs and ADSCs-Exo. Specifically, ADSCs may activate non-classical pyroptotic pathways through direct cell-cell contact, whereas ADSCs-Exo likely exert anti-inflammatory effects by encapsulating and delivering regulatory molecules. To validate these hypotheses, a co-culture system can be established to elucidate cell type-dependent effects, comparing the influence of ADSCs and ADSCs-Exo on GSDMD-N expression in primary hepatocytes and macrophages. Additionally, RNA-seq and proteomic analyses can be employed to identify ADSCs-Exo-specific miRNAs or proteins, facilitating the screening of potential molecular regulators of GSDMD. These investigations will provide critical insights into the precise roles of ADSCs and ADSCs-Exo in pyroptosis regulation and highlight potential therapeutic targets for clinical intervention. Efficacy validated in a miniature pig model of hepatectomy combined with hepatic IRI demonstrated that ADSCs treatment restored serum lactate dehydrogenase (LDH) to near-baseline levels within 72 h post-IRI (Wang et al., 2023a). In translational applications, Chen et al. (2022) developed an IL-1 receptor-targeted ADSCs subpopulation (ASCL) that synergistically combines IL-1 receptor antagonist release with inflammasome inhibition, offering significant therapeutic advantages. This approach provides a novel strategy for optimizing the targeting and efficacy of existing stem cell therapies.

### 3.2 Cardiac IRI: synergistic strategies for inflammatory microenvironment remodeling and myocardial regeneration

#### 3.2.1 Pathologic mechanisms of myocardial IRI

IRI, a key driver of ischemic heart disease progression (Tsai and Sun, 2024), involves a complex multicellular inflammatory response. This response includes neutrophil infiltration, imbalanced macrophage polarization, and programmed cardiomyocyte death, with neutrophil-mediated inflammatory cascades acting as major pro-injury factors (Algoet et al., 2023). Reperfusion triggers danger signals, such as high mobility group box 1 (HMGB1), which activate toll-like receptor (TLR) signaling and the complement system, promoting C-C motif chemokine ligand 2 (CCL2)/monocyte chemoattractant protein-1 (MCP-1)-dependent neutrophil recruitment (Frangogiannis, 2014; Natorska et al., 2023). Activated neutrophils exacerbate injury by disrupting myocardial extracellular matrix (ECM) structures through myeloperoxidase (MPO)-ROS systems and elastase-mediated degradation of laminin. Additionally, neutrophils promote microthrombosis via neutrophil extracellular traps (NETs), intensifying microvascular obstruction and perpetuating a pathological cycle of injury (Natorska et al., 2023; Rezkalla and Kloner, 2002).

#### 3.2.2 Regulatory effects of ADSCs and exosomes

ADSCs achieve multi-targeted intervention in the inflammatory microenvironment of cardiac IRI through spatiotemporal-specific regulation. ADSCs induce macrophage polarization toward the anti-inflammatory, tissue-reparative M2 phenotype, enhancing phagocytosis of apoptotic neutrophils. This reduces inflammatory injury and promotes myocardial repair, particularly in the early reperfusion phase (Zhang et al., 2020). ADSCs-exosomes deliver specific microRNAs (miRNAs) that form a sophisticated regulatory network. Notably, miR-210 promotes cardiomyocyte survival by targeting protein tyrosine phosphatase 1B (PTP1B) and reducing caspase-3/7 activity (Song et al., 2021). Similarly, miR-224-5p modulates the thioredoxin-interacting protein (TXNIP)/caspase-1/GATA4 axis and upregulates the Bcl-2/Bax ratio, decreasing cardiomyocyte apoptosis (Mao et al., 2021). Additionally, miR-221/222 exert dual protective effects by suppressing pro-apoptotic protein expression via the p38/nuclear factor kappa B (NF-κB)/p53 upregulated modulator of apoptosis (PUMA) and BCL2/adenovirus E1B 19 kDa protein-interacting protein 3 (BNIP3)/microtubule-associated protein 1 light chain 3B (LC3B)/PUMA pathways (Lee et al., 2021; 2025).

Rat model experiments demonstrated that ADSCs significantly enhance cardiac function post-IRI in acute myocardial infarction models, as evidenced by improved left ventricular ejection fraction, reduced end-systolic volume, and attenuated cardiac fibrosis (Moon et al., 2022). This integrated “three-in-one” approach—combining exosomes-mediated miRNA delivery, macrophage phenotype modulation, and apoptotic cell clearance—overcomes limitations of conventional anti-inflammatory therapies, offering a novel strategy for the clinical management of myocardial IRI. Concurrently, endoplasmic reticulum (ER) stress manifests as structural expansion and dysfunction, further aggravating hepatic injury (Jiao et al., 2020).

### 3.3 Renal IRI: interaction of antifibrotic and EPO signaling axis

#### 3.3.1 Pathologic mechanisms of renal IRI

Renal IRI is a major contributor to acute kidney injury (AKI) and chronic kidney disease (CKD) (Bonventre and Yang, 2011; Venkatachalam et al., 2015). Its core pathologic feature is microvascular pericyte-to-myofibroblast transdifferentiation, which synergizes with hypoxia-driven fibrotic cascades to promote renal fibrosis. Unlike other organs, pericytes in renal IRI differentiate into myofibroblasts that express α-smooth muscle actin (α-SMA) and secrete excessive extracellular matrix (ECM), driving collagen deposition (Humphreys et al., 2010; Liu, 2011). This process is exacerbated by chronic hypoxia, which triggers hypoxia-inducible factor (HIF) activation due to microvessel collapse and aberrant stimulation of pro-fibrotic signaling pathways, including transforming growth factor-β (TGF-β)/Smad and Wnt/β-catenin (Falke et al., 2015; Henderson et al., 2020; Humphreys et al., 2010; Liu, 2011). The local inflammatory response amplifies renal IRI pathology. During the ischemic phase, injured endothelial and tubular epithelial cells release pro-inflammatory cytokines (e.g., TNF-α], interleukin-6 [IL-6], TGF-β) and chemokines (e.g., monocyte chemoattractant protein-1 [MCP-1], regulated on activation, normal T cell expressed and secreted [RANTES]). These mediators recruit immune cells, promoting differentiation into pro-fibrotic M1 macrophages, which further enhance ECM deposition and exacerbate fibrosis (Liu, 2011).

#### 3.3.2 Regulatory effects of ADSCs and exosomes

ADSCs target the pathological network of renal IRI through precise paracrine mechanisms. ADSCs-derived vesicles mitigate IRI-induced apoptosis by downregulating microRNA-122-5p (miR-122-5p) expression, which upregulates the transcriptional activity of EPO, a target gene, and increases the Bcl-2/Bax ratio (Cheng et al., 2022). Concurrently, ADSCs upregulate SRY-box transcription factor 9 (SOX9) expression, promoting proliferation of renal tubular epithelial cells (TECs) and inhibiting their transition to a pro-fibrotic phenotype. This also indirectly suppresses aberrant myofibroblast activation by modulating the TGF-β1/Smad3 signaling pathway, reducing pathological ECM deposition. Notably, SOX9 regulation is critical, as its gene silencing significantly diminishes ADSCs efficacy. Additionally, ADSCs induce macrophage polarization toward the anti-inflammatory, pro-reparative M2 phenotype, attenuating inflammation-driven fibrosis (Ishiuchi et al., 2023; Zhu et al., 2017).

Histologic analysis of the rat renal IRI model revealed a significant reduction in renal interstitial fibrosis following ADSCs intervention. However, Ishiuchi et al. reported no significant improvement in serum creatinine levels, suggesting that ADSCs-mediated repair primarily targets tissue structure rather than immediate renal function markers (Ishiuchi et al., 2023). Recent studies further demonstrate that ADSCs from various anatomical sites exert comparable antifibrotic effects via exosomes, broadening the potential cell sources for clinical applications (Ishiuchi et al., 2023).

### 3.4 Brain IRI: combined intervention of ferroptosis blockade and microglia reprogramming

#### 3.4.1 Pathological mechanisms of cerebral IRI

Ischemic stroke ranks as the third leading cause of mortality among adults globally. Current clinical management primarily relies on intravenous administration of tissue plasminogen activator (t-PA). However, its narrow therapeutic window, contraindications, and risk of cerebral hemorrhage limit its applicability, with only 1%–2% of patients benefiting from this treatment (Ginsberg, 2008; Goldstein and Rothwell, 2010). IRI in the cerebral vasculature is a hallmark of ischemic stroke, profoundly influencing treatment outcomes and prognosis. The core pathological mechanism of cerebral IRI involves a vicious cycle of ferroptosis, neuroinflammation, and glial scarring. The brain’s high lipid content (∼50% of dry weight) lowers its resistance to ferroptosis, enabling IRI to trigger iron overload, which drives ROS bursts in lipids. This is exacerbated by a sharp decline in GPX4 activity, culminating in widespread ferroptosis (Wang et al., 2024). In the acute phase, IRI rapidly activates microglia, predominantly M1-type, releasing pro-inflammatory mediators such as TNF-α and nitric oxide (NO), which amplify neuroinflammation (Hu et al., 2012). Ferroptosis not only directly causes neuronal and glial cell death but also intensifies microglia activation, perpetuating the inflammatory cascade (Stockwell et al., 2017). This “ferroptosis-inflammation storm” ultimately promotes astrocyte scarring, forming physical barriers that impede neurite outgrowth and hinder neural repair (Patabendige et al., 2021; Xu et al., 2020). Current t-PA therapy, which solely targets thrombus dissolution, fails to interrupt this pathological network, underscoring the need for integrated strategies addressing ferroptosis, microglial polarization, and scar formation.

#### 3.4.2 Regulatory effects of ADSCs and exosomes

ADSCs ameliorate cerebral IRI by disrupting the ferroptosis-neuroinflammation-glial scarring cycle via a multidimensional strategy. ADSCs-exosomes inhibit ferroptosis through miR-760-3p-mediated suppression of CHAC1-driven GSH degradation while enhancing GSH synthesis via the Fxr2/Atf3/Slc7a11 pathway, thereby fortifying M2 microglial resistance to ferroptosis (Wang et al., 2024). Microglial polarization is reprogrammed by ADSCs-secreted miR-30d-5p, which reduces the pro-inflammatory M1/M2 ratio and attenuates neuroinflammation (Jiang et al., 2018). Autophagy activation is achieved via two mechanisms: pigment epithelium-derived factor (PEDF), a neuroprotective 50 kDa glycoprotein from ADSCs, elevates LC3-II, reduces p62, and improves autophagic flux (Huang et al., 2018), while circAkap7 sequesters miR-155-5p to relieve ATG12 inhibition, triggering ATG12-dependent autophagy to protect neurons (Xu et al., 2020). Glial scarring is suppressed by ADSCs transplantation, which curbs reactive astrocyte activation and mitigates their inhibitory effects on neural regeneration and synaptic plasticity (Jiang et al., 2014). Preclinical studies confirm that ADSCs therapy reduces infarct volume and enhances neurological recovery, underscoring the therapeutic promise of combined ferroptosis inhibition, glial scar blockade, and inflammation-oxidation axis modulation (Jiang et al., 2014). However, single-cell-level exploration of astrocyte activation and neural repair mechanisms remains critical for advancing this integrated approach.

A rat model of cerebral artery occlusion demonstrated that ADSCs transplantation significantly reduces cerebral infarct volume and enhances neurological function (Jiang et al., 2014). These findings suggest that an integrated strategy combining ferroptosis inhibition, glial scar blockade, and modulation of the inflammation-oxidation axis may offer superior clinical potential. However, the mechanisms underlying astrocyte activation and neural repair require further exploration at the single-cell level.

### 3.5 Mechanism analysis and regeneration strategies for IRI in specialized organs

#### 3.5.1 Testicular IRI: preserving reproductive function with melatonin-ADSCs combination therapy

Testicular IRI is characterized by a complex pathological network involving oxidative stress, inflammatory cascades, and fibrosis. Combination therapy with melatonin and ADSCs significantly reduces oxidative stress markers, including NADPH oxidase 1 (NOX-1), NOX-2, and oxidized proteins, while restoring testicular redox homeostasis. This synergistic intervention also potently inhibits fibrosis, as evidenced by decreased deposition of transforming growth factor-beta (TGF-β) and Smad3. Histological analysis of the rat model revealed that combination therapy significantly reduces seminiferous tubule structural damage scores, increases tubular diameter, and enhances the populations of Sertoli and interstitial cells compared to monotherapy (Chen et al., 2021). These findings underscore the cascading protective effects across the seminiferous epithelium. However, the study did not assess genetic stability during the spermatogenesis cycle (∼58 days in rats) or correlate spermatogonial recovery with sperm count and motility metrics (Zhang et al., 2023). Future research should prioritize evaluating Sertoli cell tight junction remodeling, a critical component of the blood-testis barrier, and Leydig cell testosterone secretion kinetics to confirm comprehensive restoration of reproductive endocrine function.

#### 3.5.2 Spinal cord IRI: blood-spinal cord barrier reconstruction and endoplasmic reticulum stress modulation

Spinal cord IRI is a severe complication of aortic aneurysm repair or spinal surgery, often resulting in permanent neurological deficits. The pathological progression of SCIRI unfolds in three stages: (1) Early phase (0–6 h): Ischemic shock degrades tight junction proteins (ZO-1, occludin), disrupting the blood-spinal cord barrier (BSCB) and triggering a complement C3a-mediated inflammatory storm. (2) Mid-term phase (6–24 h): Irreversible endoplasmic reticulum (ER) stress activates the PERK-eIF2α-ATF4 and IRE1α-XBP1 pathways, markedly increasing neuronal apoptosis. (3) Late phase (>24 h): Sustained inflammation drives M1-type microglia polarization, exacerbating neuronal damage, while BSCB leakage of fibrinogen induces neutrophil extracellular trap formation (NETosis), perpetuating an inflammation-thrombosis cycle. The mouse spinal cord IRI model as well as the *in vitro* OGD/R modeling study found that ADSCs-exosomes disrupt this cycle through a dual mechanism: (1) BSCB reconstruction: ADSCs-exosomes restore expression of tight junction proteins (ZO-1, claudin-5, occludin), partially reconstructing BSCB ultrastructure. (2) ER stress modulation: By delivering tumor necrosis factor-stimulated gene-6 (TSG-6), ADSCs-exosomes activate the PI3K/AKT signaling pathway while selectively downregulating ER stress proteins (GRP78, p-PERK, IRE1α, ATF6), reducing neuronal apoptosis (Lu et al., 2022).

Based on the above information, to systematically compare these organ-specific mechanisms, molecular targets, and therapeutic outcomes, Table 1 summarizes the core pathological features, ADSC intervention targets, key molecular pathways, and efficacy metrics for IRI repair across the liver, heart, kidney, brain, testicular and spinal cord.

**TABLE 1:** Key targets and efficacy of ADSCs in regulating IRI in different organs.

Organ	Core pathological features	ADSCs intervention targets	Key molecular mechanisms	Efficacy
Liver	Mitochondrial fission-pyroptosis axis vicious cycle	• Mitochondrial dynamics proteins • ADSCs-exo→NLRP3-ASC-Caspase1 complex and TLR4/MyD88/NF-κB pathway • mPTP opening	• Fission protein DRP-1/Fis-1↓,fusion protein OPA1/MFN1/2↑ • Pyroptosis-related proteins GSDMD/IL-1β/IL-18↓ • PGE2-GSK3β-dependent mPTP closure	LDH returns to baseline (72 h) ALT/AST↓ Hepatocyte necrosis and sinusoidal congestion alleviated
Heart	Neutrophil Extracellular Traps massive release	• M2 phenotype macrophages • miR-210→PTP1B • miR-224–5p→TXNIP/caspase-1/GATA4 • miR-221/222→p38/NF-κB/PUMA pathway and BNIP3/LC3B/PUMA pathway	• Enhanced clearance of apoptotic neutrophils • Caspase-3/7 activity↓ • Bcl-2/Bax ratio↑	LVEF increases, ESV decreases Fibrotic area reduced
Kidney	PGE2-GSK3β-dependent mPTP closure	• miR-122–5p/EPO axis • TGF-β1/Smad3 pathway • Sox9	• Bcl-2/Bax ratio↑ • Promotion of renal tubular epithelial cells (TECs) proliferation • Inhibition of abnormal activation of myofibroblasts	Renal interstitial fibrosis area reduced Serum creatinine improvement not significant
Brain	Ferroptosis-neuroinflammation positive feedback loop	• miR-760–3p→CHAC1 • Fxr2→Atf3/Slc7a11 • circAkap7→miR-155–5p • miR-22–3p, miR-30d-5p→M1/M2 microglia polarization imbalance	• GSH synthesis↑ • PEDF activates LC3-II autophagic flux • ATG12-dependent autophagy pathway↑ • miR-22–3p promotes M2 polarization (M1/M2 ratio↓) • Reactive astrocytes overactivation↓	Infarct volume decreased Neurological score improved
Testis	Blood-spinal cord barrier disruption-endoplasmic reticulum stress	• NOX-1/NOX-2, oxidized protein • TGF-β/Smad3 pathway	• Oxidative stress marker ROS↓ • Fibrosis biomarker TGF-β1/p-Smad3 expression↓	Improved seminiferous tubule morphology score↑ Enhanced spermatogenic microenvironment Testicular fibrosis alleviated
Spinal cord	Blood-spinal cord barrier disruption-endoplasmic reticulum stress	• ZO-1, Occludin • TSG-6→PI3K/AKT pathway	• ZO-1, Occludin expression↑, enhancing BSCB integrity • Endoplasmic reticulum stress key proteins GRP78/p-PERK/IRE1α/ATF6↓	BSCB leakage reduced Neuronal apoptosis reduced Motor function improved

* ALT/AST, Ratio of aspartate aminotransferase to alanine aminotransferase; LVEF/ESV, Ratio of Left ventricular ejection fractions to End- Systolic Volume.

## 4 Clinical translational bottlenecks and interdisciplinary optimization pathways

**FIGURE 2:**
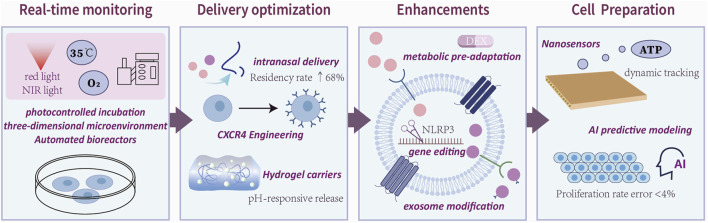
Interdisciplinary technical routes for clinical translation of therapies for ADSCs.Clinical translation of ADSCs therapies follows a four-stage pathway: cell preparation, delivery optimization, functional enhancement, and real-time monitoring. The figure reveals the main areas of concentration of current research in each area.

ADSCs hold significant promise for treating IRI, yet clinical translation faces systematic challenges requiring interdisciplinary solutions. ADSCs undergo a proliferation period of several weeks between extraction and transplantation, a culture process that carries potential risks of microbial contamination and apoptosis. Light-controlled technologies (e.g., 660 nm red light to accelerate proliferation and 415 nm blue light to regulate growth) in combination with low-temperature hypoxic culture improve proliferation efficiency and cell survival, thus addressing the limitations of the culture process (Gimble et al., 2011; Tirza et al., 2020; Wang et al., 2017; Zhou et al., 2020). While conventional cell delivery methods are inefficient, resulting in approximately 90% of cells being lost from the injection site, leakage, and poor migration, intranasal or intrathecal delivery has been applied by virtue of being more effective and less invasive, thereby enhancing ADSCs lesion targeting and improving delivery efficiency (Chung et al., 2012; Wang et al., 2023b). In addition, due to the harsh microenvironment at the site of injury (e.g., oxidative stress, inflammation), more than 80%–90% of transplanted cells will die within the first week after injection. The use of biomaterial scaffolds (e.g., chitosan-based [CSCI] hydrogels, renal extracellular matrix hydrogels) and adjuvant drugs such as dexmedetomidine may reduce the high mortality rate of grafted cells, thereby improving the retention, survival, and function of ADSCs (Cheng et al., 2022; Gao et al., 2012; Zhou et al., 2020). Genetic modification and transformation of adipose stem cell-derived exosomes can effectively enhance the targeting specificity and anti-inflammatory and antioxidant effects on specific cells, and improve the efficiency of the action of adipose stem cells. It has also been partially reported that exosomes circumvent the risk of promoting stem cell-associated tumorigenesis and immunogenicity, and will be applied in more and more disease models in the future. Modified exosomes, such as M2pep-modified exosomes for inflammatory site targeting and ADSCs-exosomes loaded with TNFα nanoparticles to enhance perfusion in ischemic tissues, are expected to enable precise multimodal therapies (Leong et al., 2020; Wang et al., 2024). Detailed information and more optimization strategies are detailed in Table 2. Collectively, these optimizations outline a phased translational pathway (Figure 2). However, rigorous validation of safety and long-term efficacy remains imperative to advance clinical application.

**TABLE 2:** Combination therapy strategies based on ADSCs/ADSCs-exosomes.

Form	Strategy/Mlecular/Drugs	Organs/Tissues/Cells	Effect
Chemical/physical treatment	Red (660 nm) or near-infrared (810 nm)	Human adipose stem cells	Stimulates the proliferation of human adipose stem cells
	Reduce the incubation temperature	Human/rat adipose stem cells	Reduces reactive oxidative substances and apoptosis in cells
	anoxia	Human adipose mesenchymal stem cells	Apoptosis decreases and the expression of angiogenesis, vascular endothelial growth factor, and basic fibroblast growth factor increases
Medication management	Dexmedetomidine (DEX)-MV	Rat NRK-52E cells	DEX-MV has a stronger ability to migrate than MV and further reduces H/R-induced cell death and ROS levels
	Hyaluronic acid (HA) + MV	IR rat model	Enhanced regenerative capacity of MSCs, increased cell proliferation and recruitment, modulation of cell-cell interactions and cell matrix adhesion
Engineering Retrofit	MSCs 3D spheres	Human adipose mesenchymal stem cells/kidney	3D globules of MSCs showed stronger anti-apoptotic and antioxidant capacity and increased paracrine secretion in MSCs
	CSCl hydrogel as MV injectable scaffold	IR renal rat model	Effectively removes reactive oxygen species and is biodegradable.It also significantly improves graft cell retention and survival
	Renal extracellular matrix hydrogel (ECMH) as an injectable scaffold	IR kidney rat/white rabbit model	It can significantly reduce oxidative stress and apoptosis, and promote cell migration, proliferation, secretion, and differentiation
Genetic modification	PrPCOE-ADMSCs	IR renal rat model	Overexpression of PrPC can enhance cell viability, proliferation, and growth of cultured ADMSCs, and enhance the ability of ADMSCs to downregulate oxidative stress and inflammation
	M2pep-ADSC-Exo	IR brain rat model	Exhibits significant targeting specificity for M2 microglia, further inhibiting ferroptosis in M2 microglia
Delivery method	Intranasal administration	IR brain rat model	IN administration is more effective than intravenous administration and less invasive than local stereotactic injection
	Intrathecal administration	IR brain white rabbit model	Minimally invasive, intrathecal ASCs may respond to signals from the damaged central nervous system

## 5 Discussion and perspectives

ADSCs and their exosomes exhibit multifaceted regulatory potential in IRI repair. ADSCs promote tissue regeneration by modulating cell differentiation, angiogenesis, inflammatory responses, apoptosis, ferroptosis, pyroptosis, and redox homeostasis (Jiao et al., 2019; Piao et al., 2022; Wang et al., 2024; Wang et al., 2023a; Zhang et al., 2021). In organs such as the liver, heart, kidney, and brain, their protective effects encompass maintaining mitochondrial homeostasis, remodeling the inflammatory microenvironment, inhibiting fibrosis, and reprogramming microglia (Jiang et al., 2018; Zhang et al., 2020; Zhang et al., 2021; Zhu et al., 2017). Despite these promising mechanisms, clinical translation faces challenges, including culture standardization, delivery efficiency, and post-transplantation cell survival. Overcoming these bottlenecks requires interdisciplinary collaboration, integrating advanced technologies such as biomaterial scaffolds, targeted delivery systems, and engineered exosomes to optimize therapeutic outcomes.

Looking forward, ADSCs hold substantial promise for advancing IRI repair. At the basic research level, multi-omics technologies, including transcriptomics, proteomics, and metabolomics, will enable comprehensive analysis of ADSCs’ mechanisms, elucidating their regulatory networks across diverse organs and pathological conditions. For instance, single-cell transcriptomics can reveal ADSCs heterogeneity and dynamics within the IRI microenvironment, informing tailored therapeutic strategies. In clinical translation, interdisciplinary approaches will accelerate ADSCs applications. Advances in materials science and biomedical engineering can optimize cell delivery systems and culture techniques, enhancing therapeutic efficacy and safety. Furthermore, progress in immunology and regenerative medicine may address immune rejection and cell survival challenges, while gene editing technologies could improve ADSCs’ specificity and potency, offering novel clinical breakthroughs. Combining ADSCs with complementary therapies, such as pharmacological agents, phototherapy, or magnetic therapy, may yield synergistic effects to enhance repair. Attention to biological variability in ADSCs from different species and individual donors is crucial for developing precise, personalized treatments. However, long-term efficacy and safety remain critical challenges, necessitating rigorous validation through future studies.

**FIGURE 3:**
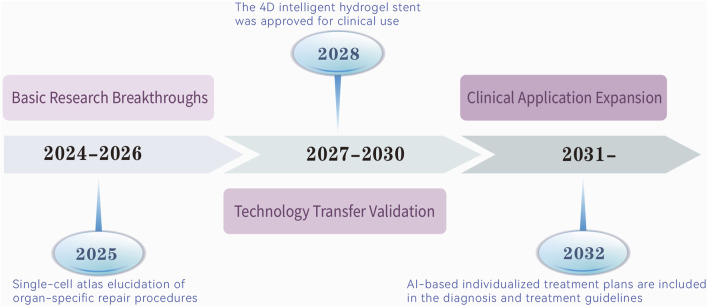
From mechanism to clinic: a 10-year route to therapeutic development in ADSCs. It predicts that ADSCs therapies will evolve in the next decade in phases from three dimensions, and reveals the key nodes in each phase.

The future of ADSCs therapy lies in a precision medicine paradigm driven by a triadic model of engineered modification, intelligent delivery, and dynamic monitoring. This approach will enable organ-specific, pathology-tailored, and biomarker-guided interventions, systematically overcoming IRI therapeutic challenges. Through interdisciplinary collaboration and technological innovation, ADSCs are poised to transition from laboratory research to clinical practice, revolutionizing organ protection strategies in intensive care settings. The transition of ADSCs therapies from mechanistic insights to clinical application necessitates a structured roadmap with defined milestones. Figure 3 delineates a ten-year development trajectory for ADSCs therapies in IRI repair, spanning three phases: basic research breakthroughs (2024–2026), marked by single-cell atlas elucidation of organ-specific repair in 2025; technology transfer validation (2027–2030), highlighted by the 2028 approval of the 4D intelligent hydrogel stent for clinical use; and clinical application expansion (2031 onward), with AI-based individualized treatment plans integrated into diagnostic and therapeutic guidelines by 2032. This roadmap underscores the progressive integration of innovative technologies to achieve clinical translation.
